# The Clinical Outcomes of a Bioinductive Collagen Implant in Bursal-Sided Partial-Thickness Rotator Cuff Tears

**DOI:** 10.3390/medicina61060988

**Published:** 2025-05-27

**Authors:** Jaesung Yoo, Daehee Lee

**Affiliations:** 1 Department of Orthopaedic Surgery, WELIVE Hoispital, Asan 31465, Republic of Korea; 2 Department of Orthopaedic Surgery, Dankook University Hospital, Cheonan 31116, Republic of Korea

**Keywords:** bioinductive collagen implant, partial-thickness rotator cuff tear, shoulder, rotator cuff repair

## Abstract

*Background and Objectives*: Many middle-aged and older individuals experience shoulder pain, often due to partial-thickness rotator cuff tears (PTRCTs). If conservative treatment fails to relieve symptoms in a patient, surgical intervention may be necessary. In such cases, using a bioinductive collagen implant may offer a viable alternative to conventional rotator cuff repair. Most notably, it offers potential advantages, particularly in reducing postoperative pain and promoting faster recovery. Accordingly, this study aims to evaluate the clinical outcomes of treating bursal-sided partial-thickness rotator cuff tears using bioinductive collagen implants alone, without concurrent rotator cuff repair. *Materials and Methods*: We followed 32 patients who had bursal-sided partial-thickness rotator cuff tears (Ellman grade I or II) and received conservative care for more than six months but continued to experience symptoms. These patients received surgery using bioinductive collagen implants without rotator cuff repair, and we followed up on their postoperative prognosis for at least one year after surgery. For a more accurate contrast, we performed clinical evaluation preoperatively and at 2 weeks, 6 weeks, 3 months, 6 months, and 12 months postoperatively. Visual Analog Scale (VAS), American Shoulder and Elbow Surgeons (ASES) score, Single Assessment Numeric Evaluation (SANE), and Western Ontario Rotator Cuff (WORC) score were used as assessment tools in this study. As for radiological outcomes, magnetic resonance imaging (MRI) and ultrasonography were helpful. This supported our assessment of graft integration and failure. *Results*: These 32 patients included 13 with Ellman grade I tear and 19 with grade II tear. In both cases, they underwent surgery only using bioinductive collagen implants, and any anchor-based cuff repair was completely excluded. As for VAS (3.8 ± 2.9), certain statistically significant improvements were found starting at 2 weeks postoperatively. On the other hand, the scores of ASES (58.6 ± 20.3), SANE (60.1 ± 23.2), and WORC (59.8 ± 22.4) began to indicate a significant improvement starting at 6 weeks postoperatively (*p* < 0.001), showing continuous progress. At each final step, we confirmed that there were no cases of graft failure by radiological evaluation and found successful healing indicators, such as much less pain in all patients. *Conclusions*: The findings of this study provide the clinical evidence that a surgery using bioinductive collagen implant for bursal-sided partial-thickness rotator cuff tears is a highly effective treatment option in patients unresponsive to conservative therapy. Particularly, its practical clinical effectiveness includes facilitating rapid recovery without a significant risk of complications.

## 1. Introduction

Partial-thickness rotator cuff tears (PTRCTs) are commonly cited as a major cause of prevalent shoulder pain in middle-aged and elderly individuals. PTRCTs also frequently result in functional impairment, considerably affecting patients’ quality of life [1]. However, such clinical presentation of PTRCTs is difficult to differentiate from other sorts of shoulder disorders due to the similarity of their symptoms, which overlap with those of other conditions (e.g., impingement syndrome and tendinopathy). Moreover, regarding imaging modalities including MRI and ultrasound, there can be various interpretations among the interobservers, which consequently leads to continual debate about optimal management strategies [2,3].

To be specific, PTRCTs are classified into articular-sided, bursal-sided, and interstitial tears based on their location. According to the Ellman classification, when tendon thickness is less than 3 mm, these are categorized as grade I tears [3,4,5,6]. Similarly, grade II tears involve tears from 3 to 6 mm, and grade III tears exceed 6 mm [4,5]. Although the best treatment for PTRCTs remains controversial, patients with tears accounting for more than 50% of the tendon thickness are often recommended to undergo a surgical intervention in clinical practice. Otherwise, their symptoms may progress to a more severe level and a higher risk of full-thickness tears [6]. However, in patients with low-grade PTRCTs (<50% thickness involvement) and particularly those experiencing constant pain despite prolonged nonoperative therapy, the optimal treatment strategy remains a significant debate [7].

For PTRCTs, many surgical alternatives such as acromioplasty, debridement, in situ repair, and completion repair have been suggested [8]. Traditional rotator cuff repair is conducted mainly by anchor-based fixation of the tendon to the humerus. But this can be accompanied by severe postoperative pain, prolonged immobilization, and increased risk of stiffness and retear [9,10]. Bioinductive collagen implants are a new treatment option that has gained attention recently in response to the above. This novel solution particularly promotes tendon regeneration while avoiding direct tendon–bone attachment. The bioinductive collagen implant (REGENETEN, Smith & Nephew, London, UK) has already demonstrated promising clinical outcomes by stimulating new tissue formation and improving tendon integrity [11].

Through prior studies, the efficacy of bioinductive collagen implants has been evaluated in various ways so far, but the studies have generally not distinguished between articular- and bursal-sided PTRCTs [12]. Considering that bioinductive implants are positioned on the bursal surface, the authors believed that the bioinductive collagen implant would be more associated with regeneration in bursal-sided partial thickness tears rather than in articular-sided partial thickness tears [11,12]. This study was designed specifically to focus on the clinical outcomes of applying a bioinductive collagen implant to bursal-sided PTRCTs without concurrent rotator cuff repair. This study hypothesized that the use of a bioinductive collagen implant for cuff regeneration in bursal-sided partial thickness tears would result in accelerated healing and superior clinical outcomes. Ultimately, this study aimed to evaluate the functional and radiological outcomes of this surgical strategy and determine its potential benefits as a middle-ground treatment between nonoperative management and traditional rotator cuff repair.

## 2. Methods

### 2.1. Study Design and Ethical Approval

This study population consisted of patients with bursal-side partial-thickness rotator cuff tears for whom conservative treatment failed for at least six months and who subsequently received surgery using a bioinductive collagen implant, excluding rotator cuff repair. The study was approved by the Institutional Review Board of Dankook University Hospital (DKUH 202503015, approved on 22 March 2025), and all participants provided written informed consent.

### 2.2. Patient Selection and Exclusion Criteria

**Figure 1:**
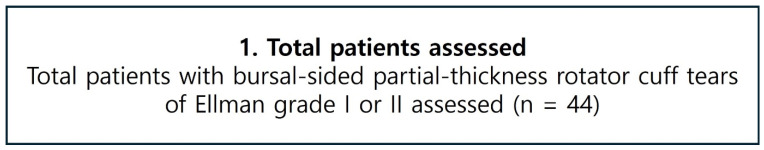
A flow diagram of patient selection. A total of 44 patients with bursal-sided partial-thickness rotator cuff tears (Ellman grade I or II) confirmed via MRI were initially assessed for eligibility. Twelve patients were excluded due to comorbid conditions, including frozen shoulder (n = 4), SLAP lesions (n = 3), osteoarthritis (n = 1), calcific tendinitis (n = 2), and distal clavicle osteolysis (n = 2). The final study cohort consisted of 32 patients who underwent treatment with a bioinductive collagen implant (REGENETEN).

In this retrospective study conducted at a single center, patients with bursal-sided partial-thickness rotator cuff tears of Ellman grade I or II confirmed by MRI were deemed eligible for inclusion. To ensure homogeneity of the study population, patients were excluded if they had articular-side tears, required rotator cuff repair, or presented with conditions such as frozen shoulder, SLAP lesions, osteoarthritis, calcific tendinitis, or distal clavicle osteolysis (Figure 1).

### 2.3. Surgical Technique

Each procedure was performed with the patient under regional block anesthesia and placed in the beach chair position, allowing optimal access to the shoulder joint. To facilitate evaluation and the use of instruments, anterior and lateral working portals were established following the initial arthroscopic examination through a posterior viewing portal.

**Figure 2:**
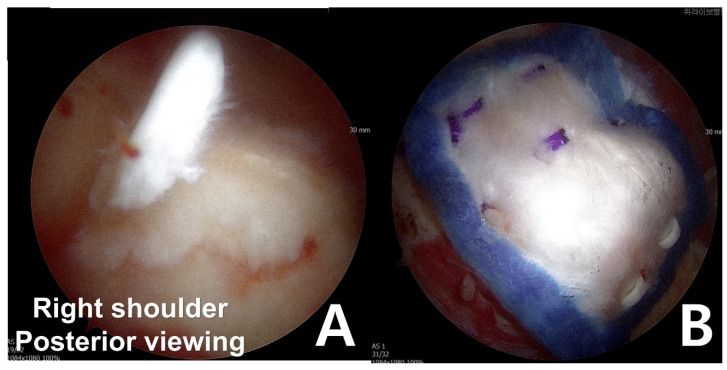
(**A**) Bursal-side partial-thickness supraspinatus tear was observed. (**B**) The bioinductive collagen implant was fixed to cover the musculotendinous junction medially and the greater tuberosity laterally.

The surgeon identified a bursal-side partial-thickness rotator cuff tear and carefully examined the lesion to rule out full-thickness involvement (Figure 2A). To preserve the native rotator cuff, frayed tendon tissue was selectively removed using a shaver and radiofrequency alation device. Afterward, the coracoacromial ligament was released and acromioplasty was performed with a burr and high-speed shaver to address the acromial spur, which contributes to subacromial impingement and tendon degeneration [13].

After establishing a superior anchor portal, a lateral working portal was created for insertion of the bioinductive collagen implant (REGENETEN, Smith & Nephew, London, UK). Using the manufacturer-provided delivery system, the implant was deployed directly onto the degenerative rotator cuff tissue and secured in position. Final arthroscopic assessment confirmed stable positioning and adherence of the implant prior to closure (Figure 2B).

### 2.4. Postoperative Rehabilitation

For the first 48 h after surgery, patients were instructed to use a shoulder sling to help protect the repair site and avoid putting stress on the implant. Once the shoulder had been immobilized for the initial period, the sling was taken off at intervals to gently begin moving the arm again, without interfering with the healing process. For the first two weeks, patients were allowed to do only passive range of motion (PROM) exercises, and movements like external rotation or lifting the arm overhead were strictly avoided.

From the second week after surgery, active-assisted range of motion (AAROM) exercises were introduced with supervision from a physical therapist, together with isometric exercises to help maintain muscle tone without overloading the healing tendon. In the weeks that followed, active motion exercises were gradually included. By the sixth postoperative week, patients were guided to achieve full active motion, taking care not to overload the joint.

At about three months postoperatively, resistance training was started using elastic bands and light weights to build strength in the shoulder. Depending on recovery progress, patients gradually resumed daily activities and sports over a period ranging from six to twelve months following surgery.

### 2.5. Clinical and Radiological Assessments

To track the clinical outcomes, functional assessments were performed before surgery and at 2 weeks, 6 weeks, 12 weeks, 24 weeks, and 52 weeks after surgery, using validated scoring tools for shoulder function. With regard to pain intensity, it was evaluated by the Visual Analog Scale (VAS). In addition, American Shoulder and Elbow Surgeons (ASES) score, the Single Assessment Numeric Evaluation (SANE), and the Western Ontario Rotator Cuff (WORC) index were used to measure patients’ shoulder performance and mobility.

**Figure 3:**
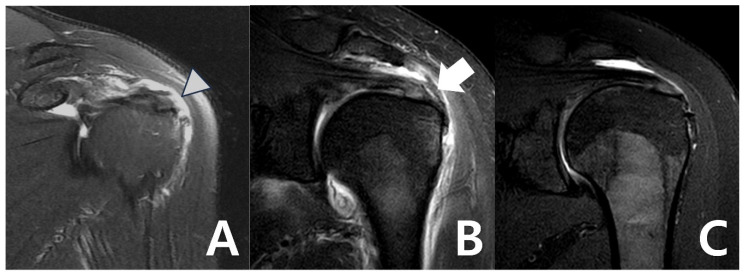
(**A**) Bursal-side partial-thickness supraspinatus tear with subacromial spur was observed on the preoperative magnetic resonance imaging. (**B**) Bioinductive collagen implant located on the tendon was observed in a postoperative magnetic resonance image. (**C**) A magnetic resonance image 6 months after surgery showed that the bio-inductive collagen implant was absorbed, and the bursal-side partial tear was fully recovered.

At 6 months and 12 months after surgery, MRI and ultrasonography were used to examine bioinductive collagen implant integration and assess the condition of the tendon tissue. MRI helped assess tendon integrity and healing, including continuity, signal changes, and possible problems, such as delamination or retear. Ultrasonography was used to offer real-time imaging of the implant’s condition (Figure 3). Radiologic assessments using MRI and ultrasonography revealed no evidence of graft failure or retear at any postoperative stage. Serial images demonstrated continuous tendon remodeling and integration of the bioinductive collagen implant into the native rotator cuff tissue. In this study, radiological evaluation after REGENETEN implantation was conducted using MRI and ultrasound findings. Regeneration was assessed based on the absence of discontinuity, fluid signal intensity, or signs of retear at the graft site on both MRI and ultrasound. Additionally, a progressive change to a homogeneous low signal on T2-weighted MRI images and a continuous, homogeneous echotexture on the ultrasound, along with an observed increase in tendon thickness of approximately 2.0 mm compared to preoperative images, were considered indicative of successful regeneration. Radiological assessments were independently performed by a board-certified radiologist with over 16 years of clinical experience.

### 2.6. Statistical Analysis

A power analysis was conducted using G*Power software 3.1.9.7. to assess the adequacy of the sample size in this study. Based on an assumed medium effect size (Cohen’s d = 0.5), a significance level (α) of 0.05, and a two-tailed test, the calculated statistical power for a sample size of 32 patients was 0.82 [14]. Statistical analysis was carried out with SPSS Statistics version 21.0 (IBM Corp., Armonk, NY, USA) on a Windows platform. A repeated measures ANOVA was utilized to analyze temporal changes in clinical outcomes. The assumption of normality was verified using the Kolmogorov–Smirnov test, which confirmed that the data were normally distributed. A *p*-value below 0.05 was considered indicative of statistical significance.

## 3. Results

### 3.1. Patient Demographics and Baseline Characteristics

This study included 32 patients who underwent arthroscopic implantation of a bioinductive collagen patch (REGENETEN) for bursal-sided partial-thickness rotator cuff tears. The mean age of the enrolled patients was 53.2 years, with a standard deviation of 9.7 years. Among the 32 patients, 18 were female and 14 were male, showing a slight female predominance. Tears were more commonly observed in the dominant arm (19 patients, 59.4%) than in the non-dominant arm (13 patients, 40.6%). The patients had an average height of 169.4 ± 12.3 cm and an average weight of 68.6 ± 10.2 kg, corresponding to a mean body mass index (BMI) of 23.7 ± 4.1 kg/m^2^.

As for health-related factors, 11 patients (34.4%) were smokers and 21 patients (65.6%) were non-smokers. Most belonged to ASA class 1 (29 patients, 90.6%), with 3 patients (9.4%) classified as ASA class 2. ASA class 3 was not observed in any case. Based on Ellman classification, 13 patients (40.6%) were classified as having grade I partial tears, and 19 patients (59.4%) as having grade II. The mean follow-up period across all patients was 14.7 months (±3.5) (Table 1).

### 3.2. Functional and Radiologic Outcomes

The Visual Analog Scale (VAS) pain scores significantly decreased after the surgical procedure, which can be interpreted as a positive impact on pain. Before surgery, the mean VAS score was 7.1 ± 4.4, which shows that patients experienced considerable pain preoperatively. Just two weeks postoperatively, it dropped to 3.8 ± 2.9 (*p* < 0.001), showing a marked early improvement. Pain scores continued to decrease over time. At 6 weeks, the mean VAS score decreased to 3.1 ± 2.7, and by 3 months, it further declined to 2.1 ± 2.2 (*p* < 0.001 for both). At 6 months, the average score reached 1.8 ± 1.9, and by 12 months, most patients reported minimal residual pain, with a final VAS score of 1.2 ± 1.6 (*p* < 0.001 for all comparisons with baseline, Table 2, Figure 3).

The American Shoulder and Elbow Surgeons (ASES) score improved significantly after surgery. Before surgery, the mean ASES score was 34.2 ± 17.5. No significant improvement was observed at 2 weeks (35.8 ± 18.2, *p* = 0.619). However, from 6 weeks onward, scores increased substantially: 58.6 ± 20.3 at 6 weeks, 78.8 ± 19.6 at 3 months, 83.3 ± 18.1 at 6 months, and 88.9 ± 17.5 at 12 months (all *p* < 0.001 compared to baseline).

The Single Assessment Numeric Evaluation (SANE) score, reflecting patients’ perception of shoulder function, also showed steady improvement throughout the follow-up period. The average preoperative SANE score was 39.5 ± 23.5. Although a slight decrease was recorded at 2 weeks (38.3 ± 20.8, *p* = 0.284), this difference did not reach statistical significance. From that point on, patients showed consistent improvement: 60.1 ± 23.2 at 6 weeks, 76.8 ± 19.8 at 3 months, 85.2 ± 21.5 at 6 months, and 90.3 ± 18.2 at 12 months (all *p* < 0.001).

**Figure 4:**
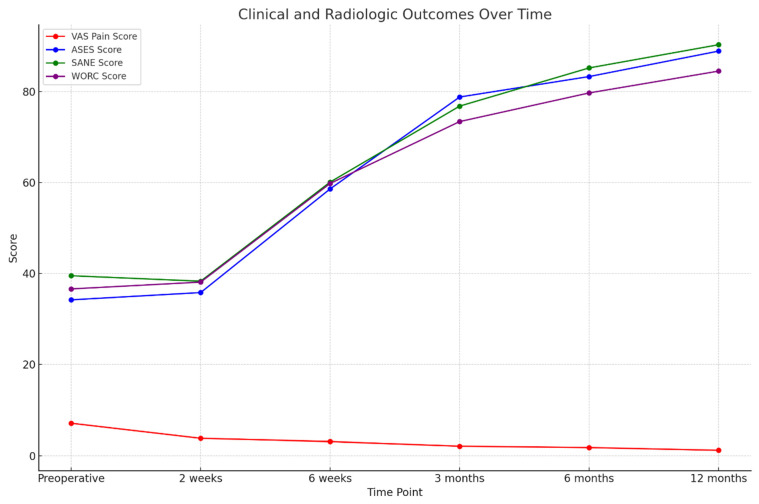
The clinical and radiologic outcomes following surgery over a 12-month period.

The Western Ontario Rotator Cuff (WORC) index, which is used to assess quality of life in patients with rotator cuff disorders, showed a similar pattern to the result mentioned above. The average preoperative WORC score was 36.6 ± 23.4, and no statistically significant change was found at 2 weeks (38.1 ± 21.8, *p* = 0.173). However, significant improvement became evident starting at 6 weeks postoperatively (59.8 ± 22.4) and continued over time, with scores increasing to 73.4 ± 23.5 at 3 months, to 79.7 ± 20.8 at 6 months, and to 84.5 ± 22.7 at 12 months (all *p* < 0.001, Table 2, Figure 4).

### 3.3. Radiologic Outcomes and Graft Integrity

Radiologic assessments using MRI and ultrasonography revealed no evidence of graft failure or retear at any postoperative stage. Serial images demonstrated continuous tendon remodeling and integration of the bioinductive collagen implant into the native rotator cuff tissue. At both 6 and 12 months postoperatively, all patients showed well-incorporated grafts with no signs of structural compromise.

## 4. Discussion

Partial-thickness rotator cuff tears (PTRCTs) are one of the most common shoulder pathologies, especially in middle-aged and older adults. These lesions can result in both functional limitations and diminished quality of life [15]. Although many PTRCTs are asymptomatic, some patients continue to experience pain and functional impairments. In such cases, surgery may be required when conservative treatment fails [16]. Among the traditional surgical options for treating PTRCTs, such as acromioplasty, debridement, in situ repair, and completion repair, many require tendon–bone reattachment using anchors, a technique that has been associated with prolonged immobilization, postoperative pain, and an increased incidence of stiffness [17].

To address these limitations, bioinductive collagen implants such as REGENETEN have emerged as a promising alternative treatment that enables the tendon to heal without requiring direct suture repair or the use of anchors [18]. Among these recent efforts, this study focused on evaluating the clinical outcomes of using a bioinductive collagen implant in patients with bursal-sided PTRCTs who were treated without tendon-to-bone fixation. The result demonstrated a significant reduction in postoperative pain, as evidenced by improved VAS scores observed at just two weeks postoperatively (*p* < 0.001). Moreover, functional outcomes, assessed by ASES, SANE, and WORC scores, showed significant improvement beginning at six weeks postoperatively (*p* < 0.001), with sustained improvement observed at later stages. These findings are consistent with earlier studies that have reported favorable clinical results using bioinductive collagen implants for rotator cuff pathology [14].

Distinguishing between articular- and bursal-sided PTRCTs is essential as they differ in both pain mechanism and healing potential—factors that directly influence treatment selection [19]. Because bioinductive implants are placed on the bursal side—where they support collagen deposition and tendon regeneration—this study focused specifically on bursal-sided tears. As a result, we confirmed their effectiveness in promoting healing in these cases of PTRCTs and supported their use as a treatment option in appropriate situations [20].

Since the subacromial bursa contains a higher density of sensory nerve endings, pain in bursal-sided PTRCTs follows a different mechanism than that in articular-sided tears. According to previous research, neural structures in the bursal layer contribute to increased pain perception in these cases, so symptom relief is now considered a key treatment goal [21]. In this study, the use of bioinductive collagen implants was found to aid in tendon healing and may help reduce pain, possibly because bioinductive collagen implants lessen the mechanical contact between the acromion and the exposed tendon. The idea that bioinductive implants can influence tendon healing has also been supported by prior investigations, which observed changes in the local mechanics of the rotator cuff [14].

A particular benefit observed with the use of bioinductive collagen implants is the way they may influence the course of postoperative rehabilitation, potentially allowing for a smoother and earlier return to activity. Patients treated with bioinductive collagen implants are able to begin rehabilitation earlier than those undergoing conventional repair. This may be because no tension is present at the site, unlike in suture–anchor constructs. As we observed in this study, early mobilization was well tolerated in all patients during the follow-up period. MRI and ultrasonography at 6 and 12 months showed no signs of implant failure or graft-related complications. Bioinductive collagen implants have been reported in previous studies to encourage tendon remodeling and integration. Unlike anchor-based techniques, no suture-related complications were observed [22].

While the results are encouraging, several limitations should be considered. This was a retrospective study, which introduces the possibility of a selection bias. In addition, potential confounding factors may have influenced the outcomes as full control over such variables was not feasible in this study design. Another limitation lies in the small sample size, which may restrict broader interpretation of the results. Still, the power analysis suggests that the sample was sufficient to identify clinically relevant changes. A further concern relates to the follow-up period (12 months), which may not have been sufficient to assess long-term outcomes, such as the durability of the bioinductive implant or the possibility of delayed failure. Thus, longer-term follow-up and randomized controlled trials will be important to confirm the sustainability of these outcomes and to refine patient selection and surgical strategies.

While most prior research did not separate articular- from bursal-sided PTRCTs, here, we looked only at the bursal ones based on the idea that the pain mechanism, including their anatomical and biomechanical characteristics, works differently in each type [2,3,4,5,6]. We also believe that the decision to perform surgery should be made not only based on imaging findings but also, and more importantly, on each patient’s persistent symptoms that do not improve with conservative care. Because distinguishing PTRCTs from early frozen shoulder remains difficult, asymptomatic partial-thickness tears are still commonly seen in older patients in daily clinical practice [8,10,13].

## 5. Conclusions

Taken together, the findings of this study suggest that bioinductive collagen implants may provide effective pain relief and functional recovery in bursal-sided PTRCTs while also supporting tendon healing and allowing for early rehabilitation. Bursal-sided tears may involve a distinct biological mechanism, which could influence healing potential. Further research will be necessary to clarify the long-term effectiveness of bioinductive implants and define their role in clinical practice.

## Figures and Tables

**Table 1:** Demographic data.

Variable	N = 32
Mean age	53.2 ± 9.7
Gender (Male:Female)	14:18
Dominant arm: Non-dominant arm	19:13
Height (cm)	169.4 ± 12.3
Weight (kg)	68.6 ± 10.2
Body mass index	23.7 ± 4.1
Smoking: Non-smoking	11:21
ASA class (1:2:3)	29:3:0
Ellman grade (1:2)	13:19
Mean follow-up (month)	14.7 ± 3.5

**Table 2:** Clinical and radiologic outcomes.

Variable	Preoperative	2 Weeks	6 Weeks	3 Months	6 Months	12 Months
VAS pain score	7.1 ± 4.4	3.8 ± 2.9	3.1 ± 2.7	2.1 ± 2.2	1.8 ± 1.9	1.2 ± 1.6
		<0.001	<0.001	<0.001	<0.001	<0.001
ASES score	34.2 ± 17.5	35.8 ± 18.2	58.6 ± 20.3	78.8 ± 19.6	83.3 ± 18.1	88.9 ± 17.5
		0.619	<0.001	<0.001	<0.001	<0.001
SANE	39.5 ± 23.5	38.3 ± 20.8	60.1 ± 23.2	76.8 ± 19.8	85.2 ± 21.5	90.3 ± 18.2
		0.284	<0.001	<0.001	<0.001	<0.001
WORC	36.6 ± 23.4	38.1 ± 21.8	59.8 ± 22.4	73.4 ± 23.5	79.7 ± 20.8	84.5 ± 22.7
		0.173	<0.001	<0.001	<0.001	<0.001
Retear, %	-	0 (0%)	0 (0%)	0 (0%)	0 (0%)	0 (0%)

VAS: Visual Analog Scales; ASES: American Shoulder and Elbow Surgeons; SANE: Single Assessment Numeric Evaluation; WORC, Western Ontario Rotator Cuff Index.

## Data Availability

The data that support the findings of this study are available from the corresponding author upon reasonable request.
